# Improved navigator based diffusion tensor MRI of the human heart in vivo

**DOI:** 10.1186/1532-429X-15-S1-W25

**Published:** 2013-01-30

**Authors:** PF Ferreira, S Nielles-Vallespin, PD Gatehouse, R de Silva, J Keegan, P Speier, T Feiweier, TG Reese, TF Ismail, A Scott, C Mekkaoui, DE Sosnovik, D Firmin

**Affiliations:** 1 Cardiovascular Biomedical Research Unit, Royal Brompton Hospital, London, UK; 2 National Heart Lung and Blood Institute, National Institutes of Health , Bethesda, MD, USA; 3 Martinos Center for Biomedical Imaging, Massachusetts General Hospital, Charlestown, MA, USA; 4 MR Application & Workflow Development, Siemens AG Healthcare Sector, Erlangen, Germany

## Background

In vivo Cardiac Diffusion Tensor Imaging (cDTI) remains extremely challenging due to a mixture of cardiac and respiratory motion. In previous work a prospective navigator technique (NAV) has been implemented and compared to breath-hold (BH) acquisitions. Statistically significant differences were found between BH and NAV techniques for helix-angle (HA) values. Further interrogation of the data suggested that the inconsistent HA patterns were found in NAV data sets in which a small fraction (up to 10%) of the diffusion-weighted frames had signal voids in some part of the LV. The purpose of this work was to improve the robustness of the NAV technique.

## Methods

A stimulated-echo single-shot-EPI sequence was used, together with a modification of the crossed slice prospective-navigator technique combined with a biofeedback mechanism. To prevent bulk respiratory motion artefacts the first and second heartbeat have to be within 1mm of each other as well as being inside the navigator acceptance window. 7 volunteers were scanned, with both BH and NAV techniques (b value=350s/mm2, 8 averages). Post-processing was enhanced by firstly adding an interface where bad frames where rejected, and secondly by using an image registration cross-correlation algorithm prior to averaging. The cDTI data was then processed to create fractional anisotropy (FA), mean diffusivity (MD) and HA maps in the myocardial region. A comparison of the BH data was then made between the two sets of NAV data: NAVold (without frame rejection and image registration) and NAVnew (with frame rejection and image registration). We specifically looked at the average myocardial FA and MD value, and the HA difference to a statistically averaged HA map from 10 healthy volunteers.

## Results

**Figure 1:**
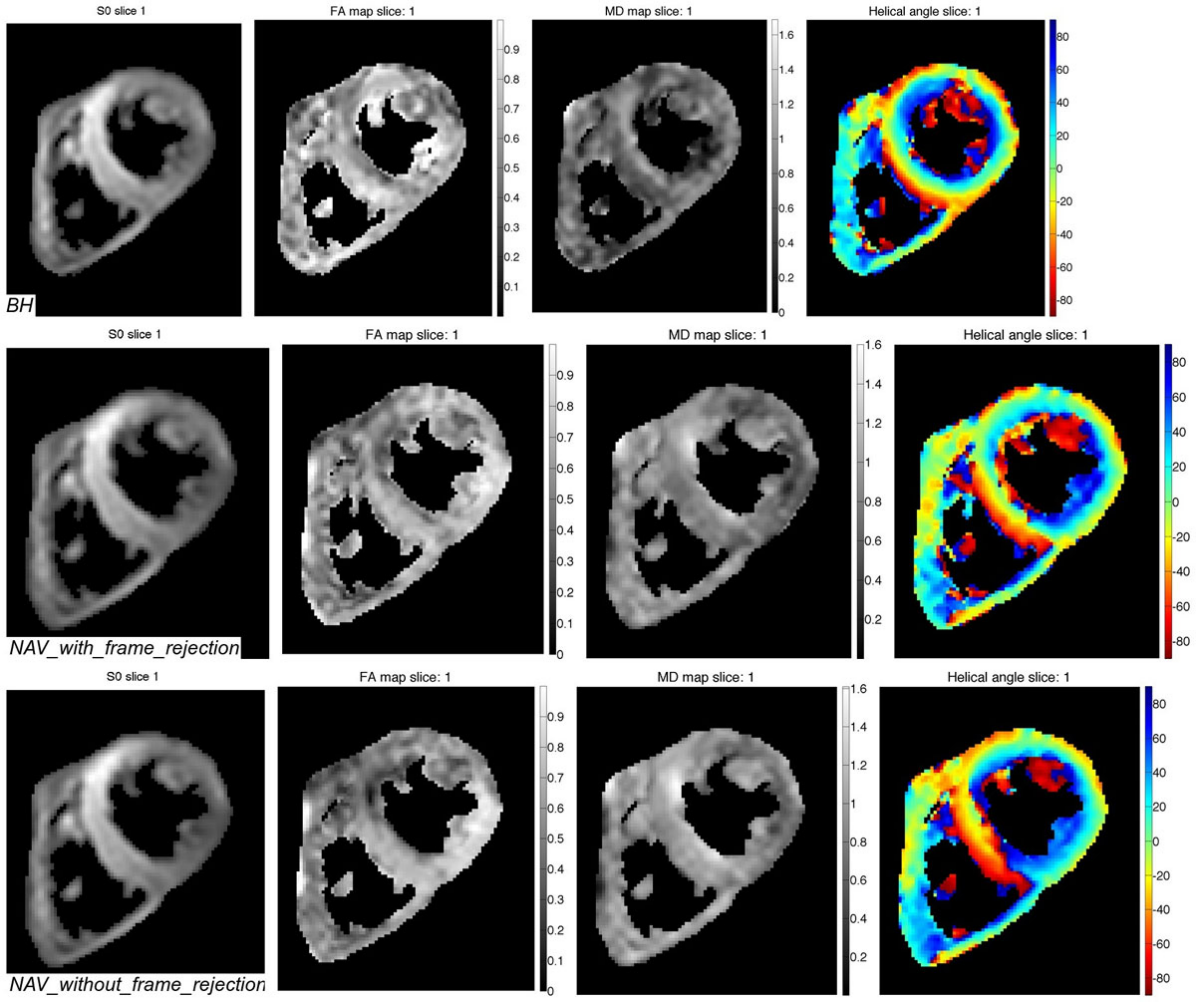
From left to right: averaged b0 image, FA, MD, and HA map. Top to bottom: BH, NAVnew, NAVold.

**Figure 2:**
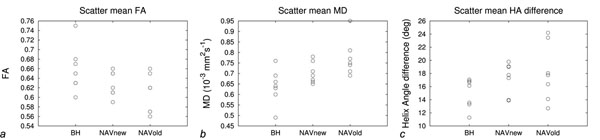
Scatter plots of the mean FA, mean MD, and mean HA difference.

A subject’s averaged b0 image, FA, MD, and HA maps are shown in Figure 1. A paired t-test of the results of all volunteers between BH and NAVold shows significant difference in the mean FA (p=0.014), and mean MD (p=0.0036) but no significant difference in the mean HA difference maps (p=0.25). No statistical difference was found between BH and NAVnew: FA (p=0.14), MD (p=0.074), and HA difference (p=0.21) (Figure 2).

## Conclusions

Here we showed for the first time that a free-breathing navigator based approach to cDTI produces high quality in vivo images, comparable to that of the BH protocol. The ability to perform free breathing DTI will be critical if the use of DTI is to be extended to patients with cardiovascular disease and limited breath-hold capacity. This could prove to be a powerful tool to characterise the structural remodelling and fibre disarray patterns of diseases such as myocardial infarction and cardiomyopathies, improving the capability of cardiac MRI for diagnosis and therapy follow-up.

## Funding

This work was supported by the NIHR Cardiovascular Biomedical Research Unit of Royal Brompton and Harefield NHS Foundation Trust and Imperial College London UK, and by the National Institutes of Health (grant: R01HL093038).

